# COVID-19 vaccination intention: The combined role of pathogen disgust and trust in government

**DOI:** 10.3389/fpsyg.2022.1024614

**Published:** 2023-01-04

**Authors:** Shun Peng, Jiwen Chen, Lei Xu

**Affiliations:** School of Education, Jianghan University, Wuhan, China

**Keywords:** COVID-19 vaccination intention, pathogen disgust, trust in government, COVID-19 worry, COVID-19

## Abstract

The present study aimed to investigate the joint effect of pathogen disgust and trust in government on COVID-19 vaccination intention and to examine the mediating role of COVID-19 worry. The data was collected from July to September 2021 in mainland China by using Questionnaire Star, 2,244 valid cases were obtained among a total of 2,251 participants investigated, with an effective rate of 89.37%. The results indicated the following: (1) Individuals’ COVID-19 vaccination intention was significantly higher when “congruence was high” than when “congruence was low”, given comparable levels of pathogen disgust and trust in government. (2) There were no significant differences in individual COVID-19 vaccination intention with incongruence levels of pathogen disgust and trust in government. (3) The combination of pathogen disgust and trust in government can influence COVID-19 vaccination intention through COVID-19 worry. Findings illustrate that individuals with high trust in government and pathogen disgust have higher intentions. Trust in government and pathogen disgust positively predicted COVID-19 worry and reinforced individuals’ intention to COVID-19 vaccination. The results have important implications for the future prevention and control of the new coronavirus, as well as providing a new perspective on COVID-19 vaccination intentions.

## Introduction

Coronavirus disease 2019 (COVID-19) is a disease caused severe acute respiratory syndrome coronavirus-2 (SARS-CoV-2) that is transmitted through aerosols and droplets (Fauci et al., 2020). In March 2020, COVID-19 was officially classified as a global pandemic (WHO, 2020). To reduce the spread of the virus and decrease the likelihood of illness, most countries recommended or required individuals to adhere to precautions such as wearing masks and vaccinations. Following the new coronavirus outbreak, the Chinese government quickly issued the Coronavirus Disease 2019 Prevention and Control Program, which advised the population to adhere to healthy behaviors such as frequent hand washing, wearing masks, and vaccination (National Health Commission of the People’s Republic of China (NHCPRC), 2021) to curb the spread of the virus (Galasso et al., 2020). A major challenge currently facing governments is how to make the population comply with these recommendations (Tominey, 2020). Studies have shown that there are still significant differences in health behaviors and vaccination intentions among people in different regions and countries (Anderson et al., 2020). In addition, the current emergence of new coronavirus variants (e.g., Delta and Omicron), even in countries with high population vaccination rates, is requiring booster shots to reinforce the “Great Wall of Viral Defense.” Therefore, it is essential to understand individual COVID-19 vaccination intentions and the factors influencing them. Previous studies have explored many variables influencing vaccination intention, such as age may be an important factor in individual health behaviors during COVID-19, as older adults face a greater risk of serious complications from COVID-19 than younger adults (WHO, 2020). In addition, women place more emphasis on health behaviors than do men (Galasso et al., 2020). The purpose of this study was to explore the relationship between pathogen disgust, trust in government and COVID-19 vaccination intention.

### Congruence of pathogen disgust and trust in government and COVID-19 vaccination intention

Evolution has provided humans with two systems that are highly sensitive to anything that contains pathogens (Schaller and Park, 2011). In addition to the reactive physiological immune system, there is also a preventive “behavioral immune system” (Schaller and Park, 2011). The behavioral immune system is a complex set of cognitive, emotional, and behavioral mechanisms that ultimately help prevent the spread of pathogens in the face of recurrent infectious disease threats (Schaller, 2006), and disgust plays a central role in this system (Olivera-La Rosa et al., 2020). Tybur et al. (2009) suggest that natural selection drives disgust toward three domains: pathogen avoidance (pathogen disgust), mate choice (sexual disgust), and social interaction (moral disgust). Among these, pathogen disgust refers to a negative emotion expressed toward objects that may transmit disease (e.g., decaying food and bodily fluids; Tybur et al., 2013). Pathogen disgust acts as an affective signal of infection risk, causing individuals to develop avoidance tendencies when faced with possible COVID-19 infection (Shook et al., 2019) and to engage in self-protective behaviors (Oaten et al., 2009; Tybur et al., 2013). Numerous related studies have shown that pathogen disgust is an important facilitator of individual health behaviors (e.g., hand washing and mask wearing) and has positive implications for preventing the spread of viruses (Shook et al., 2020; Kempthorne and Terrizzi, 2021).

However, pathogen disgust does not necessarily increase vaccination intention, and some previous studies have even shown that individuals with higher pathogen disgust have more negative attitudes toward vaccines and lower vaccination intention (Clay, 2017). For example, a study by Kempthorne and Terrizzi (2021) showed that pathogen disgust was significantly and negatively associated with vaccine intention in US participants during the COVID-19 pandemic. However, a subsequent study with Finnish participants yielded the opposite result (Karlsson et al., 2022). Some researchers have suggested that the relationship between pathogen disgust and vaccination intention depends on whether the participants are sensitive to “disgust eliciting” or not. For example, Amin et al. (2017) found that parents with high pathogen disgust were significantly less hesitant to vaccinate after viewing pictures of children with measles and rubella (disgust eliciting) as opposed to before viewing the pictures. In other words, individuals with higher disgust sensitivity may be more motivated to vaccinate after exposure to disgust-eliciting stimuli. Thus, the difference in results between the Finnish participants (Karlsson et al., 2021) and the American participants (Kempthorne and Terrizzi, 2021) may be due to the difference in the level of disgust-eliciting stimuli to which they were exposed. However, it has also been suggested that this “disgust eliciting” may be ineffective or the opposite (Horne et al., 2015; Nyhan and Reifler, 2015). The effectiveness of disgust-eliciting stimuli depends on whether the “corrective information” provided is credible (Hobson-West, 2003). Mistrust of doctors, government messages, and pharmaceutical companies and science, for example, is one of the main reasons for vaccine hesitancy (MacDonald et al., 2018). During COVID-19, government releases of relevant outbreak information (e.g., postinfection mortality rates or public media images of patients with new coronavirus infections) were one of the possible disgust elicitings. Studies have shown that hesitation to vaccinate can be reduced indirectly through exposure to COVID-19 information on the Internet and traditional news media (Liu et al., 2021). Confronted with major emergencies (e.g., COVID-19, SARS, and Great Sichuan Earthquake), most media outlets in China are willing to assume public responsibility and help the government release response information to the public. Therefore, whether the release of information acts as a disgust-eliciting stimuli is critical and also depends on whether the individual trusts the government and the information it provides. If the individual believes that the information released by the government is true, then the information may elicit disgust; if not, then it will not. In other words, individuals’ COVID-19 vaccination intention may be influenced by a combination of pathogen disgust and trust in government, the specific combination of which is shown in Table 1.

**Table 1:** A combination of pathogen disgust and trust in government.

		Trust in Government	
		High	Low
Pathogen Disgust	High	“High congruence”	“Low trust and high disgust”
	Low	“High trust and low disgust”	“Low congruence”

Trust in government refers to people’s confidence or satisfaction in the performance of government (Park et al., 2015). Previous research has shown that the higher the trust in government, the more people are willing to follow government advice and engage in pro-social behaviors, such as taking precautions to avoid swine flu and getting a seasonal flu vaccination (Verger et al., 2018). A global survey of COVID-19 vaccine acceptance conducted in June 2020 found that populations in countries with high vaccine acceptance tend to have higher trust in government (Lazarus et al., 2020). After controlling for individual perceptions of vaccine safety and risk of COVID-19, a significant negative association remained between individual trust in government and vaccine hesitation (Park et al., 2021). Therefore, when individuals’ trust in government and pathogen disgust are consistently high, they tend to follow government recommendations and have higher vaccination intention (Verger et al., 2018). Individuals with high pathogen disgust may also have higher vaccination intention after being exposed to relevant information (disgust elicitation) released by the government. In contrast, when individuals’ trust in government and pathogen disgust are consistently low, they tend not to follow government recommendations (Han et al., 2021), and those with low pathogen disgust tend to adopt fewer self-protective behaviors (Cepon-Robins et al., 2021) have a relatively low vaccination intention. Based on this, it is hypothesized (H1) that individuals will have higher vaccination intention against COVID-19 when their pathogen disgust and trust in government levels are consistently high than when their pathogen disgust and trust in government levels are consistently low.

Furthermore, when an individual’s pathogen disgust and trust in government are inconsistent, there are two scenarios, namely, “low trust and high disgust” and “high trust and low disgust.” Individuals with “low trust and high disgust,” tend to fight government advice, resist vaccination, and oppose social distance and masking (Han et al., 2021). At this point, individuals with high levels of pathogen disgust may further reduce their intention to vaccinate (Kempthorne and Terrizzi, 2021). However, when individuals exhibit “high trust and low disgust,” they are more likely to adopt government recommendations (Verger et al., 2018) and have higher vaccination intention (Chua et al., 2021). At this point, a lower level of pathogen disgust does not “impede” an individual’s intention to vaccinate, resulting in a higher intention to vaccinate. Therefore, this study hypothesized (H2) that when trust in government and pathogen disgust levels are inconsistent, COVID-19 vaccination intention is higher in the “high trust low disgust” condition than in the “low trust high disgust” condition.

### Mediating role of COVID-19 worry

Examining the relationship between the independent variable and the dependent variable merely describes the size of the “black box” of the influence of the independent variable on the dependent variable, without describing the internal processes of the black box, i.e., “how” the independent variable influences the dependent variable. For individuals, public health policies/recommendations (e.g., social distancing, masks, vaccinations) have economic and psychological costs, and the effectiveness of these measures depends on the public’s COVID-19 worry (Galasso et al., 2020). Worry refers to individuals thinking about future events in an anxious or apprehensive manner (Sibrava and Borkovec, 2006). COVID-19 worry refers to individuals’ worry and concern about COVID-19 (Faisal et al., 2020). Studies have shown that COVID-19 worry is significantly and positively related to individuals’ self-protective behaviors (Shook et al., 2020). Vaccination during the COVID-19 period is often seen as a self-protective behavior (National Health Commission of the People’s Republic of China (NHCPRC), 2021). Terror management theory (Greenberg et al., 1990) suggests that humans have a meaningful defense system to manage fear when death-related thoughts are conscious (the current focus of attention). The individual’s proximal defense is activated to suppress such thoughts or to intentionally engage in healthier behaviors to ensure a longer life span and delay death into the distant future (Arndt et al., 1997). COVID-19 serves as a striking symbol of death that stimulates individual anxiety and concern, prompting individuals to adopt proximal defenses (Coelho et al., 2020), thus promoting COVID-19 vaccination intention.

On the other hand, a study by Shook et al. (2020) found that pathogen disgust was a significant positive predictor of COVID-19 worry in individuals. The behavioral immune system can be activated by environmental cues, and the more sensitive individuals are to pathogen threat, the more concerned they are about pathogen transmission and worry about the impact of the virus on themselves (Schaller and Park, 2011). In addition, trust in government also influences individuals to worry about COVID-19. According to the trust and confidence model (TCM), trust affects public perceptions of risk and plays an important role in individuals’ responses to threats (Siegrist and Zingg, 2014). Individuals with high trust in government are more likely to accept government information (Chua et al., 2021) and believe in the impact of the COVID-19 pandemic on individuals, thus making individuals worried about and concerned about COVID-19. In conclusion, the investigators concluded that the combined scenario of trust in government and pathogen disgust affects COVID-19 worry and thus COVID-19 vaccination intention. First, when individuals’ trust in government and pathogen disgust are consistently high, they are more concerned about COVID-19 and its negative effects on themselves and have stronger infection worries, thus promoting COVID-19 vaccination intention. In contrast, when individuals’ trust in government and pathogen disgust are consistently low, they are less concerned about COVID-19, making them less concerned about its effects on themselves, and thus less willing to receive the COVID-19 vaccine. Second, when individuals’ pathogen disgust and trust in government are inconsistent, individuals with “high trust and low disgust” will choose to believe government information and will be more concerned about COVID-19 and worried about the development of COVID-19, thus affecting their intention to receive the COVID-19 vaccine. In contrast, individuals with “low trust and high disgust” will overestimate the risk of infection and have a higher level of COVID-19 concern, which will promote self-protective measures. Therefore, this study hypothesizes (H3) that pathogen disgust and trust in government can influence vaccine intention through individuals’ COVID-19 worry.

## Materials and methods

### Participants

Due to the difficulty of conducting face-to-face questionnaire surveys on a large scale during the pandemic of COVID-19, this study was carried out from July to September 2021 in mainland China by using Questionnaire Star.^1^ A total of 2,511 subjects were investigated, of which 66 did not sign the informed consent, 58 participants failed to complete the questionnaire, and the data with standard score greater than 4 were excluded. Finally, 2,244 valid cases were obtained, with an effective rate of 89.37%. The demographic information of the subjects was as follows: Among all the subjects, 1911 participants had completed the vaccination, with the vaccination rate of 85.16%, including 810 males (36.10%) and 1,434 females (63.90%). In terms of educational attainment, 3 (0.13%) were at level of primary school, 9 (0.40%) at junior high school, 99 (2.94%) at senior high school, 1905 (84.89%) at college-level and 261 (11.63%) at postgraduate level. In terms of work experience, 1908 (85.03%) were students, 12 (0.53%) were temporary or migrant workers, 33 (1.47%) were manual workers or self-employed, 174 (7.75%) were general managers or professional technicians, 84 (3.74%) were middle-level managers or intermediate technicians, 21 (0.94%) were top managers or senior technical staff, and 5 (0.53%) were retirees. The mean age of the participants was 22.06 years (range from 14 to 71 years, *SD* = 7.22).

### Study variables

#### Pathogen disgust

The pathogen disgust subscale of the Three Domains of Disgust Scale developed by Tybur et al. (2009) was used. The subscale consists of 7 items (e.g., stepping on dog poop) measured on a 7-point scale (0 “not at all disgusted” to 6 “very disgusted”), which is used to assess the level of disgust of individuals toward pathogens, with higher scores indicating higher levels of disgust. Validation factor analysis showed that this subscale had good construct validity in this study (χ^2^/*df* = 6.60, CFI = 0.98, TLI = 0.97, RMSEA = 0.05). In the present study, the Cronbach’s α coefficient for this scale was 0.78.

#### COVID-19 vaccination intention

The Vaccine Behavioral Intention Scale developed by Head et al. (2020) was used. The scale consists of 2 items (e.g., How likely you are to be vaccinated against the coronavirus?) scored on an 11-point scale (0 “not at all possible” to 10 “completely possible”). The higher the score is, the higher the individual’s intention to receive vaccination. In the present study, the Cronbach’s α coefficient for this scale was 0.71.

#### COVID-19 worry

The COVID-19 worry scale was adapted from the Swine Flu inventory (Wheaton et al., 2012) to assess individuals’ worries about the spread of COVID-19. The questionnaire consists of 7 items (e.g., How likely is it that you could become infected with COVID-19?) and is scored on a 9-point scale (0 “not at all worried” to 8 “very worried”). Higher scores indicate that individuals are more concerned about the spread of the COVID-19 virus and its impact on them. In the present study, the Cronbach’s α coefficient for this scale was 0.82. In the present study, the scale had good construct validity (χ^2^/*df* = 10.65, CFI = 0.97, TLI = 0.95, RMSEA = 0.06).

#### Trust in government

The measure of trust in government was revised from the two items (e.g., I believe the government can control the coronavirus pandemic in China) developed by Sherman et al. (2021). An 11-point scale was used (0 “completely disagree” to 10 “completely agree”). Higher scores indicate higher levels of trust in government. In the present study, the Cronbach’s α coefficient for this scale was 0.71.

### Statistical analysis method

In this study, polynomial regression and response surface analysis were used to test the impact of the combined effect (congruence/incongruence) of pathogen disgust and trust in government on COVID-19 vaccination intention. According to Edwards and Parry (1993), the following formula is needed to test the relationship between the congruence of variables and other variables:


$$Z=b_{0}+b_{1}X+b_{2}Y+b_{3}X^{2}+b_{4}XY+b_{5}Y^{2}+e_{1}$$

X was assumed to be the pathogen disgust, Y was trust in government, and Z was the COVID-19 vaccination intention. In order to test the effect of congruence between X and Y, let X = Y, and the following formula was given:


$$Z=b_{0}+\left(b_{1}+b_{2}\right)X+\left(b_{3}+b_{4}+b_{5}\right)X^{2}+e_{1}$$

Therefore, on a coordinate axis with X = Y as the horizontal axis and Z as the vertical axis, we knew that the slope $a_{1}=b_{1}+b_{2,}$and the curvature $a_{2}=b_{3}+b_{4}+b_{5}$. In order to test the effect of inconsistency between X and Y, let X = −y, and the following formula was given:


$$Z=b_{0}+\left(b_{1}-b_{2}\right)X+\left(b_{3}-b_{4}+b_{5}\right)X^{2}+e_{1}$$

Similarly, we knew that the slope $a_{3}=b_{1}-b_{2}$, and the curvature $a_{4}=b_{3}-b_{4}+b_{5}$ on the axis X = −y and Z as the vertical axis.

For hypothesis H1, (*b*_1_ + *b*_2_) should be significantly positive and (*b*_3_ + *b*_4_ + *b*_5_) insignificant. For hypothesis H2, (*b*_1_−*b*_2_) should be significantly negative and (*b*_3_ − *b*_4_ + *b*_5_) insignificant.

To test hypothesis H3, the block variable approach was used, which not only does not change the coefficients of the other variables evaluated in the equation and the total explanatory rate, but also makes the direct and indirect effects of congruence/incongruence easier to explain (Zhang et al., 2011). We fellow the procedure of Edwards and Cable (2009), the basic procedure is to combine $b_{1}X+b_{2}Y+b_{3}X^{2}+b_{4}XY+b_{5}Y^{2}$ into one variable (block variable) and then block variable is taken as an independent variable to conduct mediation effect test.

According Baron and Kenny (1986), to test the mediating effect, the following formula should be tested:


$$M=i+aX+e_{2}$$

$$Y=i+bM+c^{\prime }X+e_{3}$$

When a×b is significant, the mediating effect is significant. In this study, X was block variable, M was COVID-19 worry, and Y was COVID-19 vaccination intention.

In this study, *R* (R Core Team, 2022) was used to analyze the data, rstatix package was used to conduct descriptive statistics, lavaan package was for testing polynomial regression and mediation effect, and RSA package was for visualizing the response surface.

## Result

### Common method biases control and inspection

As Podsakoff et al. (2003) suggested, this study controlled for common method biases by collecting questionnaires anonymously during actual implementation. Common method bias refers to the artificial covariation between predictor and criterion variables due to the same data sources and measuring environment, the context or characteristics of the items. Such artificial covariation can seriously confuse the results and potentially mislead the conclusions. The reason why needs to be controlled is that it is a kind of systematic error. Researchers often adopt Harman’s single-factor test for testing the common method bias for the sake of study rigorousness. The basic assumption of this method is that if there is a large amount of variation in the common method, the factor analysis will either separate out a single factor; or a common factor explains most of the variation. In this study, the explanation rate of all items on the first common factor was 26.51%, indicating that there was no serious common method bias in this study.

### Descriptive statistics

Correlation analysis showed (Table 2) that age was significantly and positively associated with COVID-19 worry and pathogen disgust; Gender was significantly and positively associated with COVID-19 worry; pathogen disgust was significantly and positively associated with COVID-19 worry and COVID-19 vaccination intention; trust in government was significantly and positively associated with COVID-19 worry and COVID-19 vaccination intention; and COVID-19 worry was significantly and positively associated with COVID-19 vaccination intention.

**Table 2:** Results of descriptive statistics and correlation of the variables.

	*M* ± *SD*	1	2	3	4	5	6
1. Gender*^α^*	0.36 ± 0.48	—					
2. Age	22.06 ± 7.22	−0.03	—				
3. Pathogen disgust	5.09 ± 1.07	−0.03	0.12^***^	—			
4. Trust in government	9.13 ± 1.43	0.03	−0.01	0.01	—		
5. COVID-19 worry	6.63 ± 1.46	0.07^*^	0.18^***^	0.21^***^	0.09^***^	—	
6. COVID-19 vaccination intention	7.81 ± 2.03	−0.01	0.02	0.09^***^	0.19^***^	0.16^***^	—
skewness	11.58	1.33	2.93	2.58	2.95	2.89	3.26

*n* = 2,244. * *p* < 0.05, *** *p* < 0.001, dummy variables with α Gender 0 (female) and 1 (male).

### Hypothesis testing results

**Figure 1:**
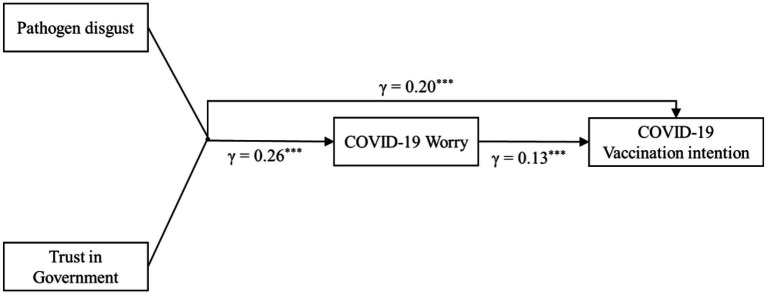
Mediation model.

To avoid multicollinearity, pathogen disgust and trust in government were centralized, followed by a multinomial regression test. Table 3 demonstrates the predictive effects of pathogen disgust and trust in government on vaccination intention and the results of the polynomial regression, which showed a significant increase in the explanatory power of model 3, with the inclusion of the squared and interaction terms of pathogen disgust and trust in government (Δ*R*^2^ = 0.01, *p* < 0.05), and compares to model 1 Δ*R*^2^ = 0.07 (*p* < 0.001), which means that response surface analysis can be performed (Nestler et al., 2019). Additionally, the slope of the consistency line (*X* = *Y*, i.e., trust in government = pathogen disgust) was tested by response surface analysis to be significantly positive (*b*_1_ + *b*_2_ = 0.43, *p* < 0.001), while the curvature was not significant (*b*_3_ + *b*_4_ + *b*_5_ = 0.01, *p* = 0.82), indicating that in terms of congruence, trust in government, pathogen disgust, and COVID-19 vaccination intention had a positive linear relationship, i.e., as the level of pathogen disgust and trust in government increased, individuals’ intention to receive the COVID-19 vaccine also increased. Additionally, regarding incongruence (*X* = −*Y*), the slope (*b*_1_ - *b*_2_ = −0.05, *p* = 0.59) and curvature (*b*_3_ − *b*_4_ + *b*_5_ = 0.05, *p* = 0.14) were not significant, indicating that under the “high trust and low disgust” and “low disgust and high trust” conditions, there was no significant difference in individuals’ COVID-19 vaccination intention (as shown in Figure 1).

**Table 3:** Polynomial regression results.

Variables	Model 1		Model 2		Model 3	
	*B*	95%CI	*B*	95%CI	*B*	95%CI
Constant (*b*_0_)	7.70^***^	[7.42, 7.98]	7.76^***^	[7.49, 8.04]	7.75^***^	[7.46, 8.02]
Gender	0.01	[−0.01, 0.02]	0.002	[−0.01, 0.01]	−0.05	[−0.22, 0.12]
Age	−0.03	[−0.21, 0.14]	−0.004	[−0.22, 0.12]	0.002	[−0.01, 0.01]
Pathogen disgust(X) (*b*_1_)			0.17^***^	[0.09, 0.25]	0.19^***^	[0.11, 0.27]
Trust in government(Y) (*b*_2_)			0.28^***^	[0.22, 0.33]	0.24^***^	[0.15, 0.33]
X^2^ (*b*_3_)					0.05^*^	[0.01, 0.10]
X × Y (*b*_4_)					−0.02	[−0.07, 0.03]
Y^2^ (*b*_5_)					−0.01	[−0.04, 0.01]
*R^2^*	0.01		0.08		0.09	
Δ*R*^2^			0.07^***^		0.08^***^	
Congruence line(X = Y)						
Slope(*b*_1_ + *b*_2_)					0.43^***^	[0.30, 0.56]
Curvature(*b*_3_ + *b*_4_ + *b*_5_)					0.01	[−0.09, 0.12]
Incongruence line(X = −Y)						
Slope(*b*_1_ + *b*_2_)					−0.05	[−0.21, 0.12]
Curvature(*b*_3_ + *b*_4_ + *b*_5_)					0.05	[−0.02, 0.14]

*n* = 2,244. * *p* < 0.05, *** *p* < 0.001, dummy variables with aGender 0 (female) and 1 (male).

### Mediation analysis

To test the mediating effect of COVID-19 worry, the raw values of five polynomials, pathogen disgust, trust in government, square of pathogen disgust, pathogen disgust × trust in government, and square of trust in government, were multiplied by the corresponding regression coefficients and summed to form a block variable. Then, the mediating effects were tested. The block variables of individual pathogen disgust and trust in government significantly and positively predicted COVID-19 worry (β = 0.26, *p* < 0.001, 95% CI = [0.22, 0.30]), the block variables of pathogen disgust and trust in government significantly and positively predicted COVID-19 vaccination intention (β = 0.20, *p* < 0.001, 95% CI = [0.16, 0.24]), and COVID-19 worry significantly and positively predicted COVID-19 vaccination intention (β =0.13, *p* < 0.001, 95% CI = [0.09, 0.17]). The mediating effect value for the block variables of pathogen disgust and trust in government affecting COVID-19 vaccination intention through COVID-19 worry was 0.03, *p* < 0.001, 95% Boot CI = [0.02, 0.05]. The mediation model is shown in Figure 1.

## Discussion

COVID-19 has caused significant negative effects, such as economic devastation, social anxiety, and human casualties, around the world. However, previous studies have found controversial effects of pathogen disgust, an important individual protective factor in times of “viral epidemics,” on vaccine intention. The present study examined the effect of matching effects of pathogen disgust and trust in government on individuals’ COVID-19 vaccination intention, and the results are a “response” to the controversies in previous studies. At the same time, the fact that approximately 1.2 billion people have been fully vaccinated in China, combined with the high level of trust in government in the descriptive statistics, is also an explanation for the current status of COVID-19 vaccination among the Chinese population.

### Relationship between pathogen disgust, trust in government, and COVID-19 vaccination intention

First, this study found that when individuals’ pathogen disgust and trust in government were consistent, individuals’ COVID-19 vaccination intention increased with increasing levels of pathogen disgust and trust in government. Hypothesis 1 was supported. Specifically, individuals’ COVID-19 vaccination intention was higher when they had higher levels of pathogen disgust and higher levels of trust in government. Solak et al. (2022) suggested that pathogen disgust is a fear of physical contamination and that individuals with high pathogen disgust are more sensitive to disease and contaminated environments, have greater uncertainty, and have a strong need for cognitive closure, so they want to make decisions as quickly as possible to eliminate this cognitive load at the expense of careful review of COVID-19 vaccines. Trust in the vaccine source/information can satisfy this need for cognitive closure and reduce uncertainty, thus making people more willing to vaccinate. This also suggests that pathogen disgust does not necessarily reduce an individual’s vaccination intention but that trust in the vaccine plays a very important role. As noted by Van Der Weerd et al. (2011), “during a pandemic, effective risk management and crisis communication are increasingly dependent on the way information is received and the level of trust in the government.”

**Figure 2:**
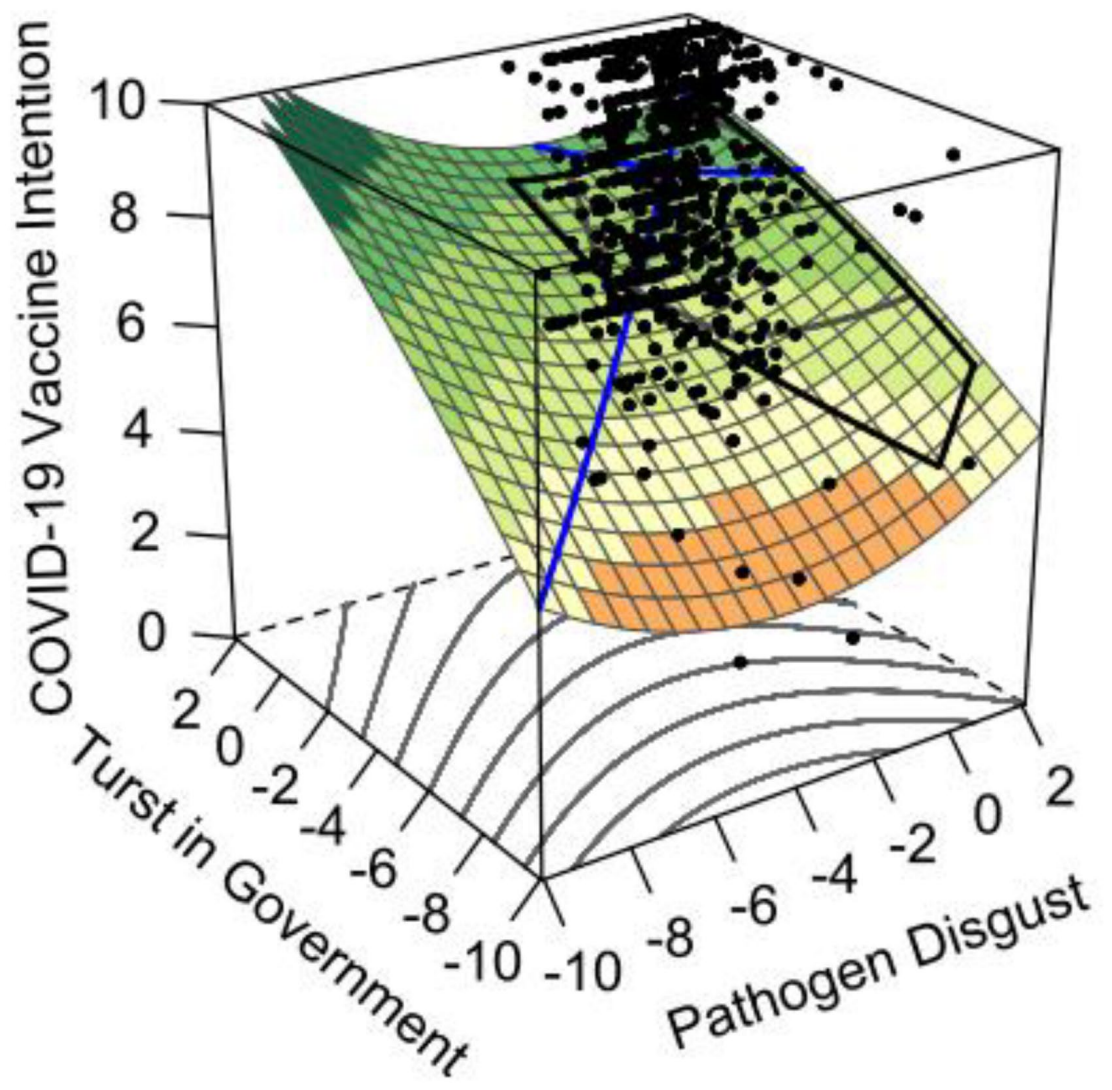
Response surface plot of pathogen disgust, trust in government, and COVID-19 vaccine intention.

Second, when individuals’ pathogen disgust was “incongruence” with trust in government, the results were not significant, instead of this study supposed that individuals’ COVID-19 vaccination intention was higher in high levels of trust in government (low levels of pathogen disgust) than in low levels of trust in government (high levels of pathogen disgust; as shown in Figure 2), Hypothesis 2 was not supported. Additionally, combined with the results of the correlation analysis, suggest that pathogen disgust in the Chinese context does not necessarily lead to low COVID-19 vaccination intention. It is probably because high disease threat reversed the relationship between the pathogen disgust and vaccination intention (Karlsson et al., 2022). For individuals with high disease threat, they believe that following vaccination has less possibility of being infected compared with SARS-CoV-2, which promotes their intention of COVID-19 vaccination. Therefore, trust in government and the pathogen disgust may play the same role in COVID-19 vaccination.

### The mediating role of COVID-19 worry

In addition, this study explored pathogen disgust, trust in government, and COVID-19 vaccination intention. The results found that COVID-19 worry mediated the relationship between pathogen disgust, trust in government, and COVID-19 vaccination intention. Hypothesis 3 was supported. The results of this study not only provide support for the relationship between pathogen disgust, trust in government, and COVID-19 vaccination intention but also reveal that COVID-19 worry can transmit the joint effect of pathogen disgust and trust in government to COVID-19 vaccination intention. In other words, the combined effect of evolved pathogen disgust and government-provided information influence individuals’ perceptions of COVID-19 in the face of the negative environment of a COVID-19 pandemic, thus guiding their behavior or behavioral intention. This study uncovers the “black box” between pathogen disgust, trust in government, and COVID-19 vaccination intention and illustrates how pathogen disgust and trust in government combine influence individuals’ vaccination intention. This study shows how pathogen disgust and trust in government combine to influence individuals’ willingness to vaccinate, which has implications for future research.

### Limitations and future directions

The findings of this study respond to previous controversies regarding the relationship between pathogen disgust and vaccination intention and illustrate the important role of the combined effect of trust in government and pathogen disgust in the process of outbreak prevention and control and vaccination. However, there are some limitations to this research. First, although there is an empirical and theoretical foundation from previous authors, this study used cross-sectional data to explore the relationship between variables, which does not indicate their causal relationship. Future studies could use longitudinal studies and experimental methods to examine the relationship between variables. Second, the subjects in this study were all from China, and the subjects’ cultural background may preclude generalization to other populations. Future studies could recruit participants from multicultural backgrounds or include factors from cultural backgrounds (e.g., collectivism, interdependent self-construal, etc.) that are unique to Chinese participants to explore. Additionally, this study failed to explore boundary conditions of the combined effecton pathogen disgust and trust in government. In fact, individual attitude also depends on affective-cognitive orientation (Di Plinio et al., 2022). It is suggested that future studies could explore the moderating effect of this variable.

## Conclusion

Two conclusions were obtained from this study: (1) Individuals with high trust in government and pathogen disgust had higher COVID-19 vaccination intention than individuals with low trust in government and pathogen disgust. (2) Trust in government and pathogen disgust positively predicted COVID-19 worries, thereby strengthening individuals’ COVID-19 vaccination intention.

## Data availability statement

The raw data supporting the conclusions of this article will be made available by the authors, without undue reservation.

## Ethics statement

The studies involving human participants were reviewed and approved by Ethics Committee of Jianghan University. Written informed consent to participate in this study was provided by the participants’ legal guardian/next of kin.

## Author contributions

SP conducted literature review, analyzed the data, participated in the data collection, improved the manuscript substantially, and wrote the first draft of the manuscript. JC generated the ideal of the study, conducted statistical analyses, and literature searches. LX and SP did vital work for the improvement of the manuscript. All authors contributed to the article and approved the submitted version.

## Conflict of interest

The authors declare that the research was conducted in the absence of any commercial or financial relationships that could be construed as a potential conflict of interest.

## Publisher’s note

All claims expressed in this article are solely those of the authors and do not necessarily represent those of their affiliated organizations, or those of the publisher, the editors and the reviewers. Any product that may be evaluated in this article, or claim that may be made by its manufacturer, is not guaranteed or endorsed by the publisher.

## Footnotes

^1^ https://www.wjx.cn/
